# On Some of the More Important Chemical Disinfectants

**Published:** 1853-02

**Authors:** George Wilson

**Affiliations:** Hon. Member of the Pharmaceutical Society of Great Britain


From the Pharmaceutical Journal (Eng.)

*On some of the more Important Chemical Disinfectants.* By George Wilson, M.D., F.R.S.E., Hon. Member of the Pharmaceutical Society of Great Britain.

The term *disinfectant*, in strictness of language, can only be applied to those agents or substances which destroy or decompose infectious or contagious matter. But it is usually employed in a wider sense, so as to include, not only *disinfectants proper*, but likewise *antiseptics* and *deodorisers*. Any attempt to draw a sharp line of demarcation between these three classes of agents, is rendered impossible by our almost total ignorance of the nature of contagious matter. Some substances, such as chlorine and sulphurous acid, possess at the same time disinfectant, antiseptic, and deodorising powers. Some, like common salt, are probably simply antiseptic; of others, such as the salts of the heavy metals, which are in high repute as deodorisers, it may be questioned whether they are of any value as disinfectants, although with some persons they rank at the head of the list. Without insisting at present on this, it may suffice to define the bodies we are about to consider, thus:—A disinfectant is an agent which effects the chemical decomposition of organic poisonous matter—the term poisonous being used in a wide sense, to include all the known or supposed causes of the development of disease, which are referred to under the names of miasma, malaria, infectious virus, contagious matter, &c.

An antiseptic is an agent which prevents or arrests the development of organic poisonous (or non-poisonous) matter, without effecting its chemical decomposition.

A deodoriser is a substance which destroys odor, by decomposing or combining with, or absorbing odorous matter. Chlorine, for example, decomposes sulphuretted hydrogen, a salt of lead also decomposes it; charcoal simply absorbs it.

Before considering the relative merits of particular substances belonging to these classes, it is necessary, however, briefly to discuss the important question: Does the poisonous organic matter which occasions certain diseases, occur in the solid, liquid, or gaseous form? The certainty that prolonged exposure to a vitiated atmosphere, such, for example, as that of a fever ward, produces disease, has led to a conclusion in which probably all concur, that the air is one of the chief media through which disease is propagated, and this connection has in turn led to the much more doubtful inference, that infectious matter is truly gaseous or vaporous. This view has probably been strengthened by the recent extensive study of the properties of anæsthetics, and by the many observations which have been made on the rapid and powerful action on the body of substances which enter it through the lungs. It has certainly also been deepened by the opinion, widely prevalent, that the gases which are evolved from cess-pools, sewers, and stagnant waters in general, particularly sulphuretted hydrogen, hydrosulphuret of ammonia, and marsh gas (light carburetted hydrogen) are the *direct* and *specific* causes of ague and fever.

If this opinion were well founded, the limits and best modes of applying disinfectants could be determined without much difficulty, and our control over infectious diseases would certainly be much greater than it is.

I think, however, that we may with confidence affirm, that the great majority of diseases are not propagated by gaseous poisons. The recent tendency to advocate an opposite opinion, has been mainly occasioned, I believe, by an opinion expressed by the late Professor Daniell to the effect, that the fatal fever of the African coast is occasioned by sulphuretted hydrogen. This view was founded on an analysis of water brought from that coast, and determined the ventilating arrangement fitted up in the vessels which formed the disastrous Niger Expedition. It appears to have been extensively adopted by medical men.

During the frequent prosecutions for nuisance, under the new police act, which took place in this city and elsewhere, during the last visitation of cholera, it occurred to me, and to other chemists, to be constantly met by endeavors on the part of the prosecutor to compel an acknowledgment that sulphuretted hydrogen, hydrosulphuret of ammonia, and marsh gas or light carburetted hydrogen, which are confessedly given off by sewage waters, are the *direct* causes of fevers and other diseases. So long as this idea prevails, and men rest satisfied with it, as the true explanation of the mode in which fevers and similar maladies originate and are disseminated, they will cease to prosecute inquiry into the matter. It is most important, therefore, to discountenance the notion that we are acquainted with the true *materies morbi.*

That neither sulphuretted hydrogen nor hydrosulphuret of ammonia produces any special disease, may be sufficiently demonstrated by the impunity with which persons are known to expose themselves to much larger quantities of these gases than can possibly act on those who suffer from exposure to marsh miasmata. In these, the nicest tests have failed to give the slightest indications of sulphuretted hydrogen, and yet a few hours' exposure to such miasms has been enough to develop fever. On the other hand, in every analytical laboratory, sulphuretted hydrogen and hydrosulphuret of ammonia are daily respired for weeks or months together by those engaged in analysis, yet analytical chemists certainly are not specially subject to fevers. At the Bonnington Chemical Works, where the ammoniacal liquor from the Edinburgh Gas Works is largely converted into sulphate and muriate of ammonia, the workmen are exposed to the hydrosulphuret of ammonia, which forms so considerable a part of the liquor; and when it is neutralized with sulphuric and muriatic acid, sulphuretted hydrogen is given off in such abundance as to blacken the silver coins and watches on the persons of the by-standers, and even (along with the carbonic acid simultaneously evolved) to render them temporarily insensible if they incautiously respire the gases. Yet no special malady is known to result from this exposure, and the Bonnington Works enjoy the reputation in the neighborhood of protecting it from the inroads of endemic and epidemic diseases. Similar observations as to the non-deleterious effects of exposure to comparatively large volumes of sulphuretted hydrogen have been made at the metal works, where a superficial tarnish of metallic sulphuret is removed by washing with acids, and the workmen are freely exposed to the sulphuretted hydrogen evolved. I need not say, that I do not wish to affirm that this gas, or its combination with ammonia, is not a powerful poison, if respired alone, or to deny that the continued entrance of either into the body, must debilitate it and prepare it for yielding to the attacks of disease. But that it is the cause of the fevers, which a very short exposure to the so-called malaria of certain districts infallibly occasions, I altogether disbelieve.

The alleged noxiousness of diluted marsh gas (light carburetted hydrogen), admits of more easy disproof, for were it the deadly agent it has been declared to be, our colliers who are exposed in coal pits to much larger volumes of it than any other class of persons, should be to a corresponding extent sufferers from the diseases which it is supposed to occasion; but unless when its mixture with air explodes, it is destitute of any injurious action on the pitmen, who are a healthy class of the community.

Another disease—namely, influenza—has been imputed, by high chemical authorities, to the diffusion through the atmosphere of a peculiar gas. Dr. Prout regarding seleniuretted hydrogen as its cause, Schonbein attributing its production to ozone. There is no evidence that either of these views is true, but much may be said in favor of the latter. The last severe epidemic of influenza spread over Europe with a rapidity which almost seems to point to a gas as the medium of its propagation. No one, however, has detected seleniuretted hydrogen in the atmosphere; and air largely impregnated with ozone may be breathed with an impunity, which throws grave difficulties in the way of Schonbein's hypothesis.

Whilst thus, with the exception of influenza (if it is to be excepted), no gas is known to possess the power of developing an infectious or contagious endemic or epidemic; on the other hand, as Professor Graham has justly remarked, such infectious matters as are accessible to us—for example, "the matter of cow-pox may be dried in the air, and is not in the least degree volatile. Indeed, the volatility of a body implies a certain simplicity of constitution, and limit to the number of atoms in its integrant particle, which true organic bodies appear not to possess. Again, the source of such bodies being at all times inconsiderable, they would, if vapors be liable to a speedy attenuation by diffusion so great as to render their action wholly inconceivable. It is more probable that matters of contagion are highly-organized particles of fixed matter, which may find its way into the atmosphere, notwithstanding, like the pollen of flowers, and remain for a short time suspended in it."

To these statements it may be added, that all chemists now acknowledge, that volatility is not essential to the transference of solid bodies to the atmosphere, at least so far as those soluble in water are concerned; for observations on the largest scale have shown that the vapors of volatile liquids carry with them sensible quantities of all the solids which they dissolve; common salt, nitrate of potass, boracic acid, phosphoric acid, afford marked examples of this; but the list of salts soluble in water, which accompany its vapor at temperatures at which when dry they are fixed, is endless.* The significant word "*Malaria*," therefore, which embodies, in a single term, the evil reputation which the air or atmosphere has acquired, as the vehicle of contagion, may still, if we choose, be retained, although we acknowledge that all accessible contagious matters are non-volatile liquids or solids. It may further be added, that with the questionable exception of influenza, no endemic or epidemic spreads with the rapidity and equability, so far as area of occurrence is concerned, which would characterise it, if it were occasioned by a gas subject to a force so pewerful as that of gaseous diffusion. Professor Graham's argument is still more cogent; for, according to his views, if infectious matters were truly gaseous, we should never have endemics or epidemics, unless those matters were developed in immensely larger quantities than by universal acknowledgement they are. In truth, they elude every test, even when applied to large volumes of the most infected atmospheres.

From all that has been stated, it must be inferred, according to our present knowledge, that at least the great majority of the substances which are intended to be reached by disinfectants, are not volatile, and therefore are much less easily decomposed than if they were gases. We may also with reasonable confidence affirm, that they are organic products, and as much consist of carbon, hydrogen, oxygen, and nitrogen, or at least of two (if not always of three) of

* In virtue of this we may anticipate the administration of other medicines than anæsthetics by the lungs, although they may not be volatile. In cases of poisoning it would be of the greatest importance, if we could directly transfer to the blood an emetic or purgative, which we may hope to do along with the vapor of its solvent, aqueous or non-aqueous. Such a process, however, would be applicable only to medicines which act powerfully in small doses.

these elements; and that like all such compounds, they are readily decomposed by chemical reagents, especially oxidizing ones. There is no reason to imagine that infectious matters are difficult to decompose, *provided we can reach them.* The difficulty lies in reaching them. Assuming then that contagious matters are not volatile, and that they contain (to take the most complex case) carbon, hydrogen, oxygen, and nitrogen, the principles which are to guide us in the application of chemical disinfectants, will not be far to seek. Oxidizing agents will plainly be of great value as they can readily convert hydrogen into water, and carbon into carbonic acid, and thus disintegrate and destroy the morbific matter. Substances having a great affinity for hydrogen, such as chlorine and its class, will plainly also be of great service. Substances having an affinity for oxygen will also be applicable to the destruction of organic poisons; and, finally, all reagents which by contact with organic matter can determine a new arrangement of its ultimate elements. All the powerful chemical disinfectants act in one or other or all of those ways. I shall refer to five of the disinfectants: 1, quicklime, including caustic potash and soda; 2, nitric acid; 3, chlorine; 4, aqua regia; 5, ozone. The value of *quicklime* and of the *caustic alkalies* as disinfectants, has certainly not been overrated, although it may be questioned whether our sanitary authorities have been wise in trusting to lime alone as a purifier. From the careful study of the process of natural and artificial nitrification, and from the results of the application of soda lime in organic analysis, we have learned that the caustic alkalies and alkaline earths decompose organic matter with the evolution of ammonia, which by oxidization may become converted into nitric acid. Woodwork or stone floors, to which a coating of limewash cannot be applied, requires only to be washed with caustic soda or soft soap, to obtain an effect identical with that which lime occasions.

2. *Nitric Acid* seems latterly to have fallen into disrepute, but certainly very undeservedly. It acts more rapidly on many organic compounds than chlorine does, attacking their carbon as well as their hydrogen, and as it is not required in large quantity its application is not costly.

(To be concluded in next No.)

